# Visualizing intraluminal duodenal diverticulum in adults: a rare clinical entity

**DOI:** 10.11604/pamj.2025.51.83.48685

**Published:** 2025-07-30

**Authors:** Hanane Aksim, Rachid Akka

**Affiliations:** 1 Hepato-Gastroenterology Department, Avicenne Military Hospital, Marrakech, Morocco

**Keywords:** Intraluminal duodenal diverticulum, epigastralgia, endoscopy

## Image in medicine

We report the case of a 72-year-old woman admitted to our clinic for etiological assessment of chronic epigastralgia resistant to usual treatments. Her medical and surgical history was unremarkable. On clinical examination, the patient was conscious, normocardiac, normotensive, eupneic, and apyretic. Abdominal examination was unremarkable, apart from a slight epigastric tenderness with no tenderness or contracture. Given this clinical picture, a laboratory work-up was carried out. It revealed a correct hemoglobin level. Leukocyte count, C-reactive protein, renal function, and lipasemia were normal. No hydroelectrolytic disorders were found, and the remainder of the laboratory work-up was unremarkable. An oeso-gastro-duodenal fibroscopy revealed two intraluminal duodenal diverticula (Panel A, arrows) in the second duodenum separated by a thin border (Panel B, arrow). Differential diagnoses included choledochal cyst, duodenal duplication, and duodenal ulcer. The diagnosis of an uncomplicated intraluminal duodenal diverticulum was made. Intraluminal duodenal diverticulum is a rare entity. It originates from a congenital malformation of the descending part of the duodenum. Incomplete recanalization of the duodenum during embryonic development is considered to lead to a pulsion diverticulum, finally resulting in a “windsock-like” configuration. Diagnosis is established by duodenoscopy and/or barium meal. Treatment is indicated in selected, symptomatic cases only. In the case presented here, the diverticulum was discovered by chance during an etiological work-up for chronic epigastralgia, and treatment was not indicated as long as the diverticulum was uncomplicated.

**Figure 1:**
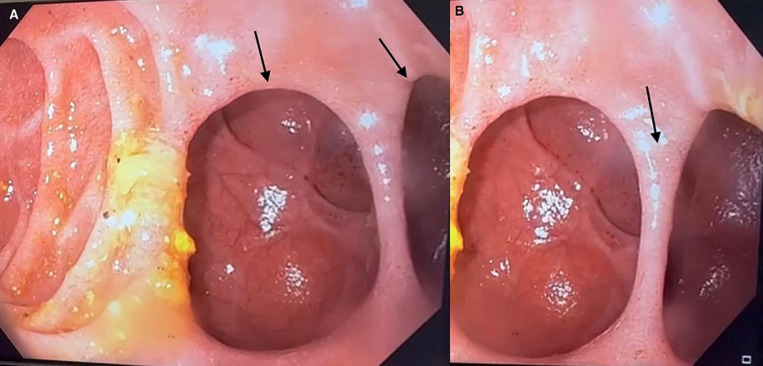
(A,B) two intraluminal duodenal diverticula in the second duodenum separated by a thin border (arrows)

